# Occult foreign body aspirations in pediatric patients: 20-years of experience

**DOI:** 10.1186/s12890-020-01356-8

**Published:** 2020-12-09

**Authors:** Bo Liu, Fengxia Ding, Yong An, Yonggang Li, Zhengxia Pan, Gang Wang, Jiangtao Dai, Hongbo Li, Chun Wu

**Affiliations:** 1 Department of Cardiothoracic Surgery; Ministry of Education Key Laboratory of Child Development and Disorders; National Clinical Research Center for Child Health and Disorders; China International Science and Technology Cooperation base of Child development and Critical Disorders, Children’s Hospital of Chongqing Medical University, No. 136, Zhongshan 2nd Road, Yuzhong Dis, Chongqing, 400014 China; 2 Chongqing Key Laboratory of Pediatrics; Chongqing Engineering Research Center of Stem Cell Therapy, Chongqing Medical University, Chongqing, PR China; 3 Department of Respiratory Medicine; Ministry of Education Key Laboratory of Child Development and Disorders; National Clinical Research Center for Child Health and Disorders; China International Science and Technology Cooperation base of Child development and Critical Disorders, Children’s Hospital of Chongqing Medical University, Chongqing, PR China

**Keywords:** Occult, Foreign body, Paediatrics

## Abstract

**Background:**

The purpose of our study was to assess the frequency of occult foreign body aspiration (FBA) and to evaluate the diagnostic difficulties and therapeutic methods for these patients.

**Methods:**

Between May 2000 and May 2020, 3557 patients with the diagnosis of FBA were treated in our department. Thirty-five patients with occult FBA were included in this study. A retrospective analysis of medical records was performed.

**Results:**

Twenty-three male patients (65.7%) and 12 female patients (34.3%) were hospitalized due to occult FBA. The average age was 3.60 years (range 9 months-12 years). Most of the patients were younger than 3 years old (*n* = 25, 71.4%). Coughing (*n* = 35, 100%) and wheezing (*n* = 18, 51.4%) were the main symptoms and signs. All the patients were found to have a FBA under the fiberoptic bronchoscope. The most common organic foreign bodies were peanuts (*n* = 10) and the most common inorganic foreign bodies were pen caps (*n* = 5). The extraction of foreign bodies under rigid bronchoscopy was applied successfully in 34 patients. Only one patient needed a surgical intervention.

**Conclusions:**

Occult FBA should always be considered in the differential diagnosis of chronic or recurrent respiratory diseases that are poorly explained, even in the absence of a previous history of aspiration.

## Background

Foreign body aspiration (FBA) is a common and serious health problem in childhood. It has a high incidence and can even be life-threatening [1]. Children under 3 years of age are most vulnerable to FBA, which is related to their narrow airways and immature protective neuromuscular mechanisms [2]. Most of them can be suspected of having a definite history of aspiration. In general, irritating cough or roaring occurs immediately after inhaling a foreign body (FB). Subsequent chronic symptoms such as cough, wheezing, stridor, fever, shortness of breath, and dyspnea often trigger the guardian’s alert so that the child can be promptly diagnosed and treated.

However, in very few cases, symptoms are mild or undetected after aspiration of the foreign body and the foreign body can stay in the bronchi for months or even years. The clinical symptoms and signs caused by foreign bodies are often not specific and the imaging signs are also not obvious. This type of FBA is difficult to distinguish from diseases such as lung infections, asthma, and congenital airway stenosis, which easily leads to missed diagnosis or misdiagnosis. Bronchoscopy is often required to detect the presence of foreign bodies. Such cases are called prolonged, suspected, or occult FBA [3–5]. Asymptomatic or long-standing occult FBA can cause irreversible complications such as bronchiectasis, bronchopleural fistula, recurrent pneumonia, lung abscess, atelectasis and even death [1].

Up to now, there is a lack of related research on children with occult FBA. In order to strengthen the understanding of its clinical characteristics, analyze diagnosis and treatment experience, and explore diagnostic ideas, the cases of occult FBA diagnosed in the Children’s Hospital of Chongqing Medical University were retrospectively analyzed.

## Methods

We retrospectively evaluated the medical records of 35 hospitalized patients who underwent bronchoscopy due to occult FBA in the Children’s Hospital of Chongqing Medical University from May 1, 2000 to May 1, 2020. The study was approved by the ethics committee of the Children’s Hospital of Chongqing Medical University (2019–48). As there is no precise definition of occult FBA, relevant literature [6, 7] was referred to, as well as combined with our own data, to define the following inclusion criteria: (1) Denies the history of FBA, (2) no typical clinical symptoms of FBA such as irritating cough; only fever, cough, wheezing and other non-typical symptoms existed, (3) no tracheal deviation was found on palpation, no tracheal tapping sound was found on auscultation, and (4) no FBs were found on radiological findings. Children with suspected FBA but negative bronchoscopy were excluded. The following individual case data were recorded:

- Age and sex,
- Course of the disease and time between the admission and the bronchoscopy,
- Chief complaint and summary of history, physical examination, laboratory tests, and radiological findings,
- Diagnosis on admission and diagnosis on discharge,
- Treatment measures,
- Endoscopic findings: FB nature, location, and complications related to FB,
- Complications related to the endoscopic procedure,
- Immediate and short-term follow up after removal.

## Results

From May 1, 2000 to May 1, 2020, 3557 patients underwent bronchoscopy and were defined diagnosed as FBA in our department. Of these patients, 35 (0.98%) met the inclusion criteria for occult FBA. 23 (65.7%) were male and 12 (34.3%) were female, and the M: F ratio was 1.92:1. The average age was 3.60 years (range 9 months-12 years). Most of the patients were younger than 3-years-old (25 patients, 71.4%). The average course was 3.69 months (range 4 days-4 years).

These patients were misdiagnosed with pneumonia, asthma, tuberculosis, and bronchitis as out-patient. Among the teaching attending rounds, the first diagnosis was pneumonia (30 cases, including 10 cases of persistent pneumonia, 4 cases of chronic pneumonia, 4 cases of severe pneumonia), 3 cases of asthma (including 2 suspected cases), 1 case of bronchitis, and 1 case of bronchiectasis. Suspected diagnoses were FBA (20 cases), tuberculosis (13 cases), asthma (11 cases), bronchiectasis (7 cases), bronchopulmonary dysplasia (6 cases), and idiopathic pulmonary hemosiderosis (2 cases).

All patients denied the history of FBA. All the 35 patients had different degrees of cough and there was no obvious tracheal deviation or tracheal tapping sounds on physical examination. The most common positive sign was wheezing (18 cases) and 7 patients had negative signs (Table 1).

**Table 1:** Characteristic of patients with occult FBA on admission

	Number	Percent
Clinical symptoms		
Cough	35	100.0
Wheezing	22	62.9
Fever	18	51.4
Shortness of breath	9	25.7
Hemoptysis	5	14.3
Dyspnea	4	11.4
Chest pain	2	5.7
Signs		
Wheezing	18	51.4
Rales	16	45.7
Asymmetry of respiratory sounds	11	31.4
Cyanosis	10	28.6
Nasal flaring / nodding breathing / Retractions	6	17.1
Negative signs	7	20.0
WBC		
< 10*10^9^/L	10	28.6
≥ 10*10^9^/L— < 15*10^9^/L	16	45.7
≥ 15*10^9^/L	9	25.7
WBC classification		
Increase in neutrophils	21	60.0
Increase in lymphocytes	14	40.0
Increased CRP	8	22.9
Sputum culture positive	14	40.0
*Streptococcus pneumoniae*	5	14.2
*Haemophilus influenzae*	3	8.6
Hemophilus parainfluenzae	2	5.7
*Staphylococcus aureus*	2	5.7
*Klebsiella pneumoniae*	1	2.9
Moraxella catarrhalis	1	2.9
Serratia marcescens	1	2.9
Viral antibody positive	6	17.1
Parainfluenza virus	3	8.6
Respiratory syncytial virus	2	5.7
Cytomegalovirus	1	2.9
Coxsackie virus	1	2.9
MP-DNA	2	5.7
Smear acid-fast and culture	0	0
X-ray		
Pneumonia	20	57.1
Atelectasis	12	34.2
Lung consolidation	10	28.6
Lung markings increased disorder	7	20.0
Pulmonary emphysema	4	11.4
Bronchiectasis	2	5.7
Pleural lesions	2	5.7
Pleural effusion	2	5.7
Mediastinal shift	1	2.9
CT scan		
Pneumonia	22	62.9
Atelectasis	15	42.9
Lung consolidation	12	34.2
Bronchiectasis	5	14.3
Invisible bronchus	5	14.3
Bronchial stenosis	4	11.4
Pulmonary emphysema	4	11.4
Enlarged or increased mediastinal lymph nodes	4	11.4
Pleural effusion	2	5.7
Bronchopulmonary dysplasia	1	2.9
Mediastinal shift	1	2.9

In laboratory tests, the results of blood routine showed an increase in white blood cell (WBC) count in 25 cases, mainly with an increase in neutrophil count. C-reactive protein (CRP) increased in 8 patients. Fourteen cases were positive for sputum bacteria culture (one case of co-infection), six cases were positive for virus antibodies (one case was co-infection), and two cases were positive for the specific DNA of mycoplasma pneumoniae (MP). Sputum smear and culture of tuberculosis were negative (Table 1).

Radiological findings after admission showed that no direct signs of FB. Occult FBAs are mainly manifested in pneumonia, atelectasis, and lung consolidation on the basis of images (Table 1).

**Fig. 1:**
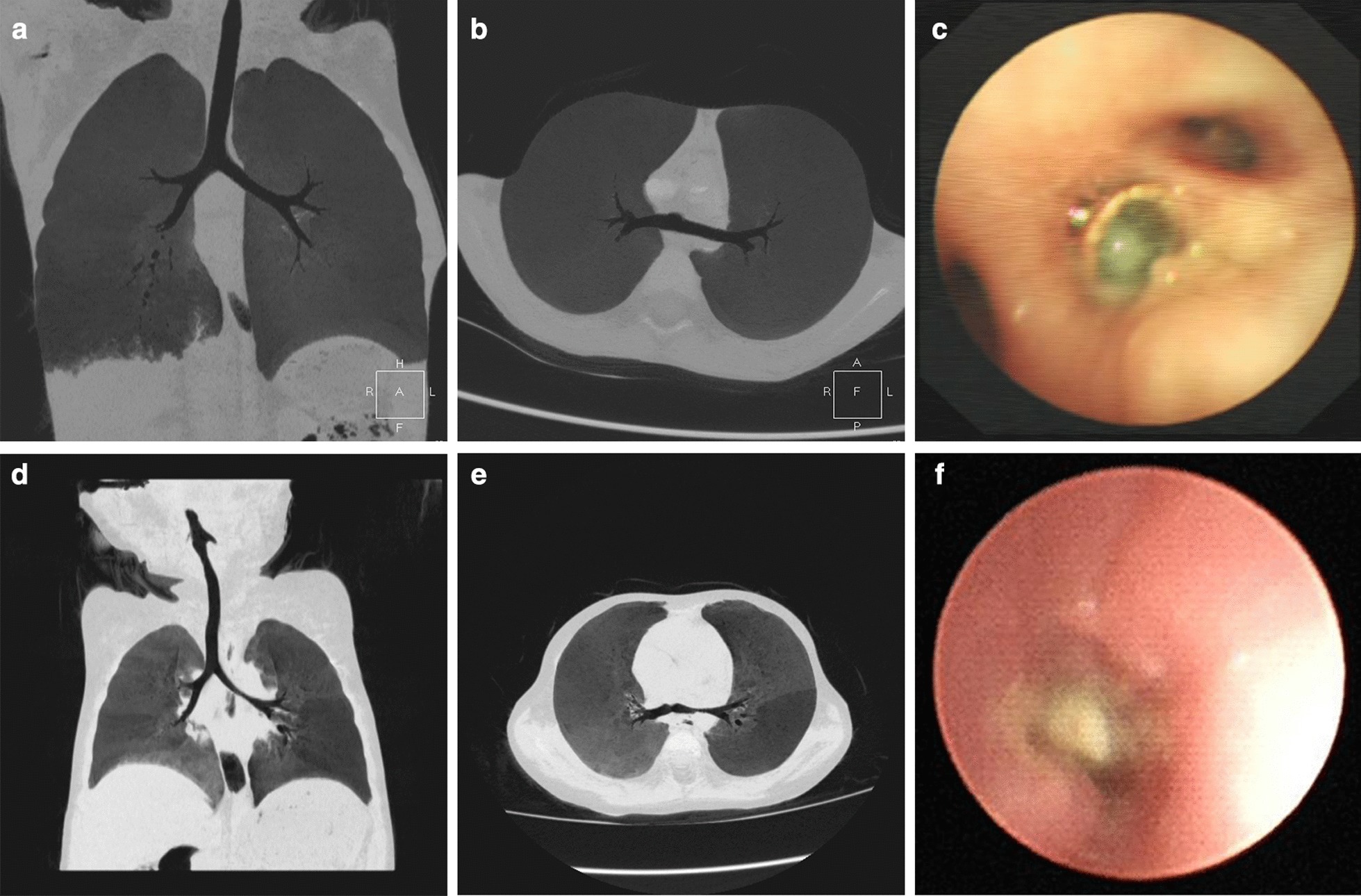
Multi-slice spiral CT (MSCT) and endoscopy of occult FBA. **a**-**c** CT showed right lower lobe lesions with bronchiectasis, and the distal bronchus was not very smooth. Clinically, sputum plugs were considered. Foreign body (red pepper) was occasionally found in the basal segment of the right lower lobe during bronchial lavage. **d-f** CT showed uneven inflation of the lungs and stenosis of the left main bronchus. We considered the presence of congenital pulmonary dysplasia. The symptoms did not ease after treatment. Endoscopy revealed a foreign body (peanut) in the left main bronchus

Thirty-five patients were treated with bronchoscopy and alveolar lavage for long-term symptoms, abnormal radiological findings, and poor treatment outcomes (Fig. 1). The time from admission to undergoing bronchoscopy for diagnosis of FBA was 1–18 days (average 3.26 days). All the patients were found to have FB under the fiberoptic bronchoscope (two cases were found by repeated fiberoptic bronchoscopy). Nineteen cases were located in the main bronchus, 12 cases were located in the lobar bronchus, and 4 cases were located in the segmental bronchus. Patients underwent rigid bronchoscopy (Karl Storz GmbH & Co KG, Tuttlingen, Germany) for FBs extraction under combined intravenous anesthesia with airway surface anesthesia. The position of FBs in 30 patients remained consistent under rigid bronchoscopy and the position changed in 5 patients. The location of the FBs in the tracheobronchial tree is shown in Table 2. After bronchoscopy, 8 patients had a transient fever, 6 patients presented as slightly irritable, and 1 patient had blood-streaked phlegm. No serious adverse events such as asphyxia, pneumothorax, dyspnea, or arrhythmia occurred.

**Table 2:** The nature of FBs and the condition of the airway

	Number	Percent
FBs location (fiberoptic bronchoscope)		
RMB	12	34.3
RUL	1	2.9
RML	3	8.6
RLL	6	17.1
LMB	7	20
LUL	1	2.9
LLL	5	14.3
FBs location (rigid bronchoscope)		
RMB	10	28.6
RUL	1	2.9
RML	2	5.7
RLL	8	22.9
LMB	7	20
LUL	0	0
LLL	7	20
Organic FBs		
Peanuts	10	28.6
Sunflower seed	5	14.3
Bones	3	8.6
Broad beans	2	5.7
Almonds	2	5.7
Pepper	2	5.7
Others	2	5.7
Inorganic FBs		
Pen caps	5	14.3
Plastic sheet	2	5.7
Plastic paper	2	5.7
Size of FBs		
< 5 mm	9	25.7
≥ 5 mm— < 10 mm	18	51.4
≥ 10 mm	8	22.9
Condition of the airway		
Granulation/ bronchial stenosis	25	71.4
Mucosal hyperemia and edema	13	37.1
Purulent secretion	8	22.9
Mucosal erosion and ulceration	3	8.6
Bleeding	1	2.9

*RMB* right main bronchus, *RUL* right upper lobe, *RML* right middle lobe, *RLL* right lower lobe, *LMB* left main bronchus, *LUL* left upper lobe, *LLL* Left lower lobe

Extracted FBs were divided into organic or inorganic types, the type was in the great majority organic (77.1%). The most common organic FBs were peanuts (*n* = 10) and the most common inorganic FBs were pen caps (*n* = 5). Because the majority of the FBs stay in the bronchial tubes for a long time, there are granulation tissue proliferation, hyperemia and swelling, and even erosion with purulent secretions in the bronchus (Table 2).

After the FBs were removed, the patient continued to be treated with anti-infective drugs and support care. The time from admission to discharge was 3–35 days (average 8.74 days). The patients had no fever before discharge and the symptoms of cough and wheezing were significantly relieved. One patient coughed up a small amount of residual FB after rigid bronchoscopy. The discharge diagnosis of all patients was FBA with pneumonia, 7 patients had bronchiectasis (including 2 suspected cases), and 5 patients had respiratory failure.

The follow-up time was 6 months – 4 years (average 2.35 years). The patients were recovered completely with full lung expansion after a mean duration of 3 months. Thirty-four children had no new pulmonary lesions in the chest image during follow-up. One patient required a pulmonary lobectomy because of bronchiectasis accompanied with lung abscess formation.

## Discussion

FBA is a common, serious, and potentially life-threatening disease occurrence in children [1]. In the present study, 71.4% of FBAs occurred around the age of 3 years. Children at this stage are at high risk of FBA due to: (1) an immature neural mechanism and poor chewing ability; (2) lack of posterior dentition; (3) the tendency to put various objects in their mouths; (4) and the habit of laughing, crying, and playing at the time of eating [8]. Boys appear to be more prone to FBA, perhaps because they are more active than girls and the M: F ratio in our study was 1.92: 1. Due to the well-known anatomic and physiological characteristics of airways, most FBs easily become lodged in the right bronchial tree [9].

The majority of occult bronchial foreign bodies are relatively small (77.1% of FBs < 10 mm in our study), it can quickly enter the main bronchus, lobar bronchus, and even segmental bronchus after aspiration, which makes it difficult to stimulate the rapid adapting irritant receptors (RARs) and causes severe irritating cough [10]. Aspiration can remain undetected and occult for a long time. The average time from onset of symptoms to admission in our study was 3.69 months. Clinical symptoms and physical findings are usually related to the size, type, and location of the FB, age of the patient, and the length of stay [11]. Presenting symptoms such as coughing, wheezing, and shortness of breath may be nonspecific. As the diagnosis of occult FBA is often missed or delayed, chronic complications such as persistent pneumonia, bronchiectasis, and abscess secondary to recurrent pulmonary infection often occur [12]. One of our patients had to undergo a lobectomy because of severe bronchiectasis with chronic empyema.

Types of FBs are closely related to religious beliefs, alimentary habits, and especially the age of the patient [13]. Organic FBs such as peanuts and sunflower seeds are often observed in children under 3 years of age, whereas inorganic FBs such as pen caps are usually found in older children. Although the inorganic FB is relatively large, it is mostly hollow or flake-shaped in our study, which makes it difficult to cause symptoms of acute airway obstruction and is not easily detected. Western cities in China are famous for their spicy food, especially hot pot. In our case, the foreign body in 2 patients was red pepper. Therefore, the relationship between alimentary habits and types of foreign bodies draws attention [14]. FBs that remain for a long time destroy the protective effect of the bronchial mucosa and increase the risk of infection from bacteria, viruses, and even mycoplasma. In response, symptoms after infection aid to find the presence of FBs.

The diagnosis of FBA depends on a high index of clinical suspicion, symptoms, signs, and radiological findings [15]. The following situations that make diagnosis difficult are easily overlooked in clinical work: (1) negligence or deliberate concealment of FBA (especially the elderly, babysitters, and people with mental illness). (2) Patients may have symptoms of lung infections such as cough and wheezing at the early stages. The imaging signs may not be obvious, and anti-infective treatment may be effective. If the child has recurrent lung infections and treatment is effective but symptoms easily reoccur then we need to highly suspect the presence of FBA. (3) Although other diseases have been definitely diagnosed, FBs may be present at the same time. One patient in our study was diagnosed with tuberculosis and antituberculosis treatment was performed concurrently. However, FBs were found during bronchial lavage. (4) FBs are more likely to fall into the right lung and lower lobe, but coughing and body position changes can cause the position of FBs in the trachea to change. In our study, five patients had different locations of FBs under rigid bronchoscopy and fiberoptic bronchoscopy. (5) Patients with bronchial comorbidities may need repeated bronchoscopy. The location of the foreign body may be too deep or the bronchial stenosis makes the foreign body difficult to reach. At the same time, granulation tissue may wrap foreign bodies, making them difficult to detect. Granulation or bronchial stenosis were present in 71.4% of our patients. (6) Even if the foreign body has been removed by the bronchoscope, we still need to be vigilant about the residue of the foreign body. One patient in our study coughed up a small amount of residual foreign body after removing the foreign body under rigid bronchoscopy.

Although fiberoptic bronchoscopy has been advocated by some researchers [16], rigid bronchoscopy is still the gold standard for removing bronchial foreign bodies in children [17, 18]. Compared with fiberoptic bronchoscope, removal of FBs under rigid bronchoscope is safer. Rigid bronchoscopes provide a clear field of vision, support continuous airway ventilation, and allow a variety of forceps to manipulate FBs. Open surgery may be required when chronic complications such as severe bronchiectasis and lung abscess has occurred. Of note, there are some special cases reported in the literature. When a patient with FBA was undiagnosed and asymptomatic for a long time, without any lung injury, the foreign body was accidentally detected, and bronchoscopy was not successful under local anesthesia. What’s the best way to deal with it? Should the patient be simply followed up or should thoracotomy be performed [19]? This case deserves our attention and reflection. In addition, our study adopts a retrospective design. There is no control group, only these who had a bronchoscopy could be detected. Therefore, the true incidence of occult FBA is still unclear, and more relevant studies are needed to clarify this problem.

## Conclusions

In conclusion, occult FBAs in children are rarely encountered in clinical practice. However, FBA should always be considered in the differential diagnosis of chronic or recurrent respiratory diseases. The most important preventive measures for foreign body aspirations are school education and family education.

## Data Availability

The datasets used and/or analysed during the current study are available from the corresponding author (BL, lbcqmu@126.com) on reasonable request.

## Abbreviations

FB: Foreign body
FBA: Foreign body aspiration
CRP: C-reactive protein
MP: Mycoplasma pneumoniae
RARs: Rapid adapting irritant receptors
WBC: White blood cell
